# AF-React study: Prevalence of thrombotic events in patients with atrial fibrillation receiving NOACs – real-world data analysis from northern Portugal primary healthcare

**DOI:** 10.3389/fmed.2024.1273304

**Published:** 2024-04-12

**Authors:** Susana Silva Pinto, Andreia Teixeira, Teresa S. Henriques, Hugo Monteiro, Carlos Martins

**Affiliations:** ^1^São Tomé Family Health Unit (ACeS Santo Tirso/Trofa), Porto, Portugal; ^2^Department of Community Medicine, Information and Health Decision Sciences (MEDCIDS), Faculty of Medicine, University of Porto, Porto, Portugal; ^3^CINTESIS@RISE - Center for Health Technology and Services Research, Faculty of Medicine, University of Porto, Porto, Portugal; ^4^ADiT-LAB, Instituto Politécnico de Viana do Castelo, Rua Escola Industrial e Comercial Nun’Álvares, Viana do Castelo, Portugal; ^5^CI-IPOP (Health Research Network), Portuguese Oncology Institute of Porto, Porto, Portugal; ^6^Regional Health Administration of Northern, Minister of Health, Porto, Portugal; ^7^#H4A Primary Health Care Research Network, Porto, Portugal

**Keywords:** atrial fibrillation, anticoagulant, prevention, stroke, primary health care, cardiovascular event

## Abstract

**Introduction:**

Anticoagulation is recommended for stroke prevention in patients with atrial fibrillation (AF). The guidelines suggest non-vitamin K antagonist anticoagulants (NOACs) as the primary therapy for anticoagulation in AF. Several patient-related factors increase the risk of thrombotic events: elderly individuals, a previous history of stroke, and chronic kidney disease. This study aims to determine the association between NOACs and other patient variables in AF and the occurrence of thrombotic events.

**Methods:**

The database included all adults with the code K78 (ICPC-2 code for AF) who received clinical care in Northern Portugal’s Primary Health Care between January 2016 and December 2018 and were dispensed the same NOAC at the pharmacy.

**Results:**

The results indicate that 10.2% of AF patients on NOAC anticoagulation experienced a stroke. Furthermore, patients treated with apixaban and dabigatran had higher odds of experiencing a stroke compared to those treated with rivaroxaban. Among patients with the same age, gender, and CHA_2_DS_2_Vasc Score, apixaban was significantly associated with a higher likelihood of thrombotic events than rivaroxaban.

**Discussion:**

These results have not been previously reported in studies with real-world data; therefore, a more detailed analysis should be conducted to enhance the validity of these findings.

## Introduction

Anticoagulation is recommended for stroke prevention in patients with atrial fibrillation (AF) (1, 2). Several novel oral anticoagulants (NOACs) have been introduced to the market due to their demonstrated efficacy, safety, and non-inferiority to Vitamin K antagonists (VKA) (3). Numerous studies have shown that NOACs outperform warfarin in preventing ischemic stroke in patients with AF, resulting in reduced major bleeding events and greater convenience of usage (4–6).

The 2020 ESC Guidelines (7) and the 2021 EHRA practical guide (8) recommend NOACs as the first-line therapy for anticoagulation in AF patients who do not have prosthetic mechanical heart valves or moderate-to-severe mitral stenosis.

The nationwide cohort study revealed no statistically significant differences in the risk of stroke or systemic embolism among NOACs, but both dabigatran and apixaban showed a significantly lower risk of major bleeding when compared with rivaroxaban. This reduction in bleeding risk associated with dabigatran and apixaban remained consistent for clinically relevant non-major bleeding and major and intracranial bleeding. Additionally, dabigatran and rivaroxaban were associated with a significantly higher risk of gastrointestinal bleeding when compared to apixaban (9). A systematic review of network meta-analyses and real-world evidence demonstrated that, in patients with AF, apixaban had comparable effectiveness to rivaroxaban and a lower risk of major bleeding events (10).

Both stroke and bleeding risks vary significantly across the spectrum of patients with AF (4).

Several patient-related factors increase the risk of thrombotic events, including elderly individuals (aged ≥75 years) (11), a previous history of stroke (12, 13) and chronic kidney disease (14, 15). A multicenter observational study conducted in seven European countries confirmed the existence of risk factors for embolic and bleeding events in anticoagulated AF patients, such as prior stroke, older age, and heart failure (16).

In the first part of the AF-React study, which utilized real-world data from the northern region of Portugal, Silva Pinto et al. (17) demonstrated that 95.8% of patients with AF indicated they received anticoagulation therapy between 2016 and 2018. Among this group, 21,854 patients (38.9%) consistently received the same NOAC [rivaroxaban, 8,801 (40.2%); apixaban, 7,052 (32.3%); dabigatran, 5,219 (23.9%); and edoxaban, 782 (3.6%)] (17). The current study aims to determine the association between NOACs and clinical variables in patients with AF and their correlation with the occurrence of thrombotic events.

## Materials and methods

### Study design and participants

A retrospective longitudinal study was conducted using the AF-React database. This project’s database included all adults (age ≥18 years) under the care of the Regional Health Administration of Northern Portugal with the code K78 (ICPC-2 code for AF) in their clinical records of primary healthcare until December 2018. For this paper, we utilized a subset of patients who dispensed the same NOAC at the pharmacy during the study period. All NOAC dispensations made by patients were considered, regardless of whether the prescription was issued by their family doctor, a hospital doctor from the National Health Service, or a doctor from a private institution.

### Ethical issues

Data processing was conducted by the Department of Studies and Planning of the Regional Health Administration of Northern Portugal under the Ministry of Health. The data were extracted from the server through an anonymized data processing and editing platform and securely delivered to the principal investigator in compliance with legal regulations and necessary approvals. The study protocol received approval from the Health Ethics Committee and Data Protection Officer of the Northern Regional Health Administration.

### Data collection

The Department of Studies and Planning of the Regional Health Administration of Northern Portugal constructed a 2016, 2017, and 2018 database using electronic health records. For this study, data were considered up to December 31, 2018, and included age, gender, professional situation, and CHA_2_DS_2_-VASc Score. The CHA_2_DS_2_-VASc Score was calculated based on the guidelines in effect during the study period (ESC guidelines in 2016 for AF) using the following ICPC-2 coding: C–congestive heart failure (K77): 1 point; H–hypertension (K86 or K87): 1 point; A_2_-age >75 years or older: 2 points; D–diabetes mellitus (T89 or T90): 1 point; S_2_-stroke (K89, K90, or K91): 2 points; V–vascular disease (K75 or K92): 1 point; A–age 65–74 years: 1 point; and Sc–sex category (female): 1 point. The glomerular filtration rate (GFR) and anticoagulant dispensation history were also available with longitudinal information and registration dates. Glomerular filtration rate (GFR) is a continuous variable not normally distributed and described by the median and respective interquartile interval, *Med (Q1; Q3)*, where *Q1* is the first quartile and *Q3* is the third quartile. The GFR was calculated automatically in the SClinico^®^ primary healthcare software by the equation of *Cockcroft-Gault* after each creatinine value record, using the last recorded weight value. To reduce the number of missing values, creatinine values were requested. Thus, GFR was also calculated by the investigators with data on weight, creatinine, age and gender, according to *Cockcroft-Gault* equation.

The cardiovascular risk in the years of 2016, 2017 and 2018 was extracted from SClinico^®^ based on the percentage of SCORE for the data that were available in the first record of 2016, 2017, and 2018, respectively. Four stages based on SCORE as low, moderate, high and very high, if less than 1%, between 1 and 5%, between 5 and 10% and greater than or equal to 10%, respectively. For users, whose SCORE does not apply, the cardiovascular risk in the years of 2016, 2017, and 2018 was calculated by the research group, according to the stages: the high and very high stages of cardiovascular risk were defined on the codification of health problems by ICPC-2 and the patient’s GFR was registered in the clinical process by the investigation group. High risk was defined in case of code T89 (Diabetes insulin dependent) or T90 (Diabetes non-insulin dependent) without other codes that are cardiovascular risk factors or if the user has a glomerular filtration rate between 30 and 60 ml/min. Very high risk was defined if the user had any of the codes K74 (Ischaemic heart disease w. angina), K75 (Acute myocardial infarction), K76 (Ischaemic heart disease w/o angina), K89 (Transient cerebral ischaemia), K90 (Stroke/cerebrovascular accident), K91 (Cerebrovascular disease) or K92 (Atherosclerosis/PVD) or if the user had code T89 (Diabetes insulin dependent) or T90 (Diabetes noninsulin dependent) with any other codes K86 (Hypertension uncomplicated), K87 (Hypertension complicated), T93 (Lipid disorder) or P17 (Tobacco abuse) or if the user had a glomerular filtration rate below from 30 ml/min.

The study also extracted comorbidity information of patients with AF.

### Statistical analysis

Categorical variables are presented as absolute and relative frequencies, *n* (%). Normally distributed continuous variables are summarized using the mean and standard deviation, M ± sd. For non-normally distributed continuous variables, the median and the respective interquartile range are reported as Med [Q1; Q3], where Q1 corresponds to the first quartile, and Q3 corresponds to the third quartile. The normality of the distributions was assessed by observing the respective histograms.

The association between categorical variables was analyzed using the chi-square test. For comparisons among more than two distributions, the One-way ANOVA test was applied when dealing with normal distributions, and the Kruskal-Wallis test was used for non-normal distributions. Multiple comparisons with Bonferroni’s adjustments were conducted in cases where significant differences were observed between the distributions. The respective effect magnitudes (ES) were determined based on the specific test used: Chi-square—Phi coefficient; Multiple comparisons—ES = Z/√(N), where Z represents the standardized test statistic, and N is the sample size. Effect magnitudes of 0.1 were considered small, 0.3 were considered medium, and values above 0.5 were classified as large.

To identify explanatory variables (drug, age, gender, CHA_2_DS_2_-VASc, CV risk in 2016, CV risk in 2017, CV risk in 2018, GFR in 2016, GFR in 2017, GFR in 2018, and comorbidities) associated with the occurrence of a thrombotic event (K89 or K90), simple logistic regressions were conducted. Among the significant variables from the simple models, age and gender were included in the multiple models as they are non-modifiable risk factors for stroke, along with the CHA_2_DS_2_-VASc Score, which is a risk score for stroke in patients with AF. The results of these regressions are presented using odds ratios (OR), respective 95% confidence intervals (95% CI), and p-values. The final model’s adequacy was assessed using the Hosmer and Lemeshow test. A significance level of *P* ≤ 0.05 was considered statistically significant. Data analysis was performed using SPSS v. 27.

## Results

Of the total 63,526 patients diagnosed with atrial fibrillation/flutter (ICPC-2 K78 code) in the northern region of Portugal, we identified 24,426 patients who obtained the same NOAC at the pharmacy during the study period. Specifically, 5,725 (23.4%) patients were dispensed dabigatran, 9,771 (40.0%) were dispensed rivaroxaban, 7,995 (32.7%) received apixaban, and 935 (3.8%) were prescribed edoxaban.

Among the variables analyzed, a statistically significant association was observed between patients receiving any of the four NOACs and several factors, including age, gender, professional status, CHA_2_DS_2_Vasc Score, CV risk in 2017, CV risk in 2018, alcoholism, smoking, ischemic heart disease with angina, cerebrovascular disease, acute myocardial infarction, hypertension artery with complications, heart failure, and previous stroke. Table 1 presents these results.

**TABLE 1:** Association between patients undergoing one of the four NOACs and patient variables.

	Rivaroxaban (*n* = 9771)	Apixaban (*n* = 7995)	Dabigatran (*n* = 5725)	Edoxaban (*n* = 935)	*p*-value and Effect size
**Age (years)**					
Med [Q_1_; Q_3_]	76 [68; 83]	79 [71; 84]	78 [71; 84]	77 [68: 83]	<0.001^a^*
**Gender, *n* (%)**					
Female	4875 (38.4)	4369 (34.4)	2957 (23.3)	494 (3.9)	<0.001^b^*
Male	4896 (41.7)	3626 (30.9)	2768 (23.6)	441 (3.8)	ES = 0.041
**Professional situation, *n* (%)**					
Active or student	2643 (44.8)	1662 (28.2)	1342 (22.7)	256 (4.3)	<0.001^b^*
Retired	6229 (37.9)	5688 (34.6)	3929 (23.9)	593 (3.6)	ES = 0.072
Not active or Unknown	899 (43.1)	645 (31.0)	454 (21.8)	86 (4.1)	
**CHA_2_DS_2_-VASc, Med [Q_1_; Q_3_]**	**3 [2; 4]**	**4 [3; 5]**	**4 [3; 5]**	**3 [2; 4]**	<0.001^a^^,^*
**RCV**					
RCV 2016, Med [Q1; Q3]	4 [3; 4]	4 [3; 4]	4 [3; 4]	4 [3; 4]	0.095^a^
RCV 2017, Med [Q_1_; Q_3_]	4 [3; 4]	4 [3; 4]	4 [3; 4]	4 [3; 4]	0.016^a^*
RCV 2018, Med [Q_1_; Q_3_]	4 [3; 4]	4 [3; 4]	4 [3; 4]	4 [3; 4]	0.017^a^*
**GFR**					
GFR 2016, Med [Q_1_; Q_3_]	68.5 [52.9; 88.0]	68.9 [52.4; 88.4]	68.8 [51.7; 86.3]	70.8 [53.1; 87.1]	0.243^a^
GFR 2017, Med [Q_1_; Q_3_]	66.6 [50.5; 86.0]	66.9 [50.2; 86.6]	67.1 [50.5; 86.5]	67.5 [49.2; 86.4]	0.946^a^
GFR 2018, Med [Q_1_; Q_3_]	65.6 [49.2; 85.7]	65.6[49.7; 85.4]	65.8 [49.5; 86.6]	66.7 [49.7; 86.2]	0.932^a^
**Comorbidities, *n* (%)**					
Chronic alcohol abuse, P15	420 (43.3)	326 (33.6)	171 (17.6)	54 (5.6)	<0.001b* ES = 0.032
Tobacco abuse, P17	519 (44.4)	363 (31.0)	246 (21.0)	42 (3.6)	0.017b* ES = 0.020
Coronary heart disease ischemic heart disease with angina, K74	398 (36.8)	420 (38.9)	221 (20.4)	42 (3.9)	<0.001b* ES = 0.029
Cerebrovascular disease, K91	329 (34.0)	368 (38.0)	246 (25.4)	25 (2.6)	<0.001b* ES = 0.031
Acute myocardial infarction, K75	289 (38.0)	287 (37.7)	154 (20.2)	31 (4.1)	0.016b* ES = 0.021
Hypertension complicated, K87	2227 (38.2)	1911 (32.8)	1482 (25.4)	204 (3.5)	<0.001b* ES = 0.029
Heart failure, K77	2110 (39.1)	1837 (34.0)	1279 (23.7)	176 (3.3)	0.012b* ES = 0.021
Stroke/cerebrovascular accident, K90	727 (33.6)	813 (37.6)	545 (25.2)	78 (3.6)	<0.001b* ES = 0.043

*Significant at 5%.

^a^Kruskal-Wallis test.

^b^Chi-square test.

In the nominal variables, the following differences were found: gender—for apixaban, dabigatran and edoxaban the female gender ratio is higher (54.6, 51.7, and 52.8%, respectively) whereas the ratio in the rivaroxaban is identical (49.9% for female and 50.1% for male); professional status—apixaban has a higher proportion of retired users (71.1%), while rivaroxaban, edoxaban and dabigatran have a lower proportion (63, 63.4, and 68.6% respectively); alcoholism—dabigatran has the lowest proportion of users (2.9%) and edoxaban has the highest proportion (5.8%); smoking—dabigatran has the lowest proportion of users (2.9%) compared to apixaban (4.5%), dabigatran (4.3%) and edoxaban (4.5%); ischemic heart disease with angina—apixaban has the highest proportion of users (5.3%) and dabigatran the lowest proportion (3.9%); cerebrovascular disease—apixaban has the highest proportion of users (4.6%) and edoxaban the lowest proportion (2.7%); acute myocardial infarction—apixaban has the highest proportion of users (3.6%) and dabigatran the lowest proportion (2.7%); hypertension artery with complications—dabigatran has the highest proportion of users (2.6%) compared to apixaban (2.4%), rivaroxaban (2.3%) and edoxaban (2.2%); heart failure—apixaban has the highest proportion of users (22.9%) compared to dabigatran (22.3%), rivaroxaban (21.6%) and edoxaban (18.8%); previous stroke—apixaban has the highest proportion of users (10.2%) and rivaroxaban the lowest proportion (7.4%).

For continuous variables, multiple comparisons with Bonferroni’s corrections were performed for these variables, and the following differences were found: Age—rivaroxaban and dabigatran (Med = 76, Med = 78, respectively; *p* < 0.001; ES = −0.08), rivaroxaban and apixaban (Med = 76, Med = 79, respectively; *p* < 0.001; ES = −0.10), edoxaban and dabigatran (Med = 77, Med = 78, respectively; *p* < 0.001; ES = 0.05) and edoxaban and apixaban (Med = 77, Med = 79, respectively; *p* < 0.001; ES = 0.06); CHA_2_DS_2_-VASc Score—edoxaban and dabigatran (Med = 3, Med = 4, respectively; *p* < 0.001, ES = 0.05), edoxaban and apixaban (Med = 3, Med = 4, respectively; *p* < 0.001, ES = 0.05), rivaroxaban and dabigatran (Med = 3, Med = 4, respectively; *p* < 0.001, ES = −0.07) and rivaroxaban and apixaban (Med = 3, Med = 4, respectively; *p* < 0.001, ES = −0.08). It is verified that the effect size of all associations is small.

Following the diagnosis of atrial fibrillation/flutter (ICPC-2 K78 code), 2,492 (10.2%) patients experienced a stroke (ICPC-2 K89 or K90 codes). Comparing the odds of stroke occurrence among patients treated with different NOACs, it was found that individuals receiving apixaban and dabigatran had statistically significantly higher odds of experiencing a stroke compared to those on rivaroxaban (OR = 1.38, 95% CI = [1.25; 1.53], *p* < 0.001; OR = 1.26, 95% CI = [1.13; 1.40], *p* < 0.001, respectively). Additionally, with respect to age, it was observed that older patients had higher odds of having a stroke (OR = 1.04, 95% CI = [1.03; 1.04], *p* < 0.001).

Being female was significantly associated with higher odds of experiencing an event (OR = 1.11; 95% CI = [1.02; 1.21]; p = 0.013). Moreover, patients with higher CHA_2_DS_2_Vasc scores were significantly associated with increased odds of having an event (OR = 3.63, 95% CI = [3.47–3.80], *p* < 0.001).

Regarding pathologies, dyslipidemia (OR = 1.25, 95% CI = [1.15; 1.36], *p* < 0.001), insulin dependent diabetes (OR = 1.56, 95% CI = [1.20; 2.01], p < 0.001), non-insulin-dependent diabetes (OR = 1.11, 95% CI = [1.01; 1.22], *p* = 0.033), ischemic heart disease with angina (OR = 1.17, 95% CI = [0.97; 1.42], *p* = 0.107), cerebrovascular disease (OR = 7.02, 95% CI = [6.13; 8.04], *p* < 0.001), acute myocardial infarction (OR = 1.73, 95% CI = [1.42; 2.11], *p* < 0.001), hypertension with complications (OR = 2.86, 95% CI = [2.62; 3.11], *p* < 0.001) and heart failure (OR = 1.26, 95% CI = [1.15; 1.39], *p* < 0.001) are clinical conditions that were significantly associated with higher odds of have a stroke. This simple logistic regression analysis of the association between the several variables and the thrombotic event risk (ICPC-2 K89 or K90) is described in Supplementary Appendix A.

In the multiple logistic regression analysis (Table 2), it is observed that the model does not exhibit a good fit (Hosmer and Lemeshow: *p* < 0.001). However, when considering patients with the same age, gender, and CHA_2_DS_2_Vasc Score, apixaban was found to be significantly associated with higher odds of experiencing an event compared to rivaroxaban (OR = 1.32, 95% CI = [1.16; 1.50], *p* < 0.001).

**TABLE 2:** Multiple logistic regression.

Multiple logistic regression		
	**OR 95% CI**	***p*-value**
**Drug**		
**Rivaroxaban**	**Reference**	
Apixaban	1.32 [1.16; 1.50]	<0.001
Dabigatran	1.17 [1.01; 1.35]	0.037
Edoxaban	1.31 [0.97; 1.76]	0.074
**Age**	**0.91 [0.90; 0.92]**	**<0.001**
**Gender**		
**Male**	**Reference**	
Female	0.16 [0.14; 0.18]	<0.001
**CHA_2_DS_2_-VASC score**	**6.11 [5.75; 6.50]**	**<0.001**

## Discussion

This study presents three main findings. First, among AF patients on NOAC anticoagulation, 10.2% experienced a stroke. Second, patients treated with apixaban and dabigatran had higher odds of stroke (statistically significant) than those treated with rivaroxaban. Third, for patients of the same age, gender, and CHA_2_DS_2_Vasc Score, apixaban was significantly associated with a greater likelihood of experiencing a thrombotic event relative to rivaroxaban.

Our results reveal a statistically significant association between each of the four NOACs and patient variables, including age, gender, professional status, CHA_2_DS_2_Vasc, CV risk in 2017, CV risk in 2018, alcoholism, smoking, ischemic heart disease with angina, cerebrovascular disease, acute myocardial infarction, hypertension artery with complications, heart failure, and previous stroke. However, these associations exhibit a small effect size, possibly attributed to the substantial sample size. While some studies have explored the association of patient variables with the risk of stroke, we did not find any studies that assess the association of the four NOACs with the various patient variables.

In our study, 10.2% of AF patients on NOAC anticoagulation experienced a stroke, a result consistent with findings in the literature. A population-based retrospective cohort study conducted in France in 2015 reported a stroke incidence of 10.1 (95% CI: 9.6–10.6) per 1000 person-years in AF patients newly treated with NOACs (18). Another study indicated that 1.0–2.0% of individuals with AF who receive one of the novel oral anticoagulants (dabigatran, rivaroxaban, or apixaban) each year could expect to experience an acute ischemic stroke (19). These outcomes suggest the potential influence of other patient-related factors contributing to the increased stroke risk, warranting a more detailed analysis of independent predictors of stroke risk.

Our results indicate that apixaban and dabigatran were associated with higher odds of stroke (statistically significant) compared to rivaroxaban. In a multivariate model, we confirmed that, for patients with the same age, gender, and CHA_2_DS_2_Vasc Score, apixaban was significantly linked to a greater likelihood of experiencing an event relative to rivaroxaban. However, a large registry-based study, which included 65,563 anticoagulant-naive patients with AF initiating OAC therapy, found no statistically significant differences in the risk of stroke between dabigatran, rivaroxaban, and apixaban (20). These variations may be attributed to the fact that the study conducted by Rutherford et al. (9) considered patients who initiated oral anticoagulants during the study period, while our study included patients who had been on the drug for an extended period. On the other hand, the pharmacokinetic characteristics of each drug differ, with an impact on bioavailability. Thus, the bioavailability of dabigatran (3% to 7%) is substantially lower than that of other NOACs (50% for apixaban, 62% for edoxaban and 66% for rivaroxaban). It is also important to highlight that the bioavailability of rivaroxaban increases to approximately 100% when taken with food, with no influence on dabigatran and apixaban and minimal interference with edoxaban. Regarding renal clearance, it is 80% for dabigatran, 50% for edoxaban, 35% for rivaroxaban, and 27% for apixaban, while non-renal clearance is, respectively, 20, 50, 65, and 73%. With regard to hepatic metabolism, it appears that there is an influence of CYP3A4 on the hepatic elimination of rivaroxaban and apixaban, although it is non-existent for dabigatran and minor for edoxaban. Simultaneous use with proton pump inhibitors (PPIs) or H2 receptor antagonists leads to a decrease in the bioavailability of dabigatran, without producing any effect on the remaining NOACs. Apixaban and rivaroxaban can also be crushed and used in nasogastric tubes, without compromising bioavailability, unlike dabigatran, whose capsules cannot be opened (21, 22). On the other hand, apixaban and dabigatran are drugs that are taken twice a day, while rivaroxaban and edoxaban are taken once a day, which promotes patient adherence to therapy. Although we analyzed data on drug dispensing in pharmacies, there may be the possibility of patients forgetting to take medication, which is more likely to occur with twice-daily drugs (apixaban and dabigatran). This means they have no anticoagulant effect for a temporary period and increase the risk of stroke. Furthermore, the criteria for dose reduction for each of the four molecules are different, possibly contributing to prescription mistakes. Therefore, stroke prevention may be compromised if a suboptimal dose is prescribed in patients without indication for dose reduction. Besides, we compared patients taking all four available NOACs (dabigatran, rivaroxaban, apixaban, and edoxaban), whereas Rutherford et al. (9) compared dabigatran, rivaroxaban, apixaban, and warfarin, there by using different association variables. Another study comparing the safety and efficacy of apixaban vs. rivaroxaban for stroke prevention in patients with AF concluded that an increased preference for rivaroxaban was significantly associated with a higher risk of major bleeding but not stroke (23). Similarly, a study in UK general practice comparing the real-world effectiveness and safety of direct oral anticoagulants in patients with non-valvular AF to prevent stroke found that apixaban was as effective as rivaroxaban in reducing the rate of stroke (24).

Among studies conducted with real-world data, our research contributes new insights into the risk of stroke in patients with AF treated with the four NOACs, as we observe statistically significant differences in stroke risk between them. While the Hosmer-Lemeshow test did not demonstrate a good fit of the data to the model, it’s essential to note that the model’s objective is not to make predictions but rather to take an exploratory approach in identifying variables associated with the outcome.

However, we must acknowledge potential registration bias in our study, particularly related to the registration of stroke events. A stroke is an event diagnosed at the hospital level, raising the possibility of underreporting in primary healthcare. Additionally, access to data on fatal stroke events was unavailable. This bias could be addressed in the future by integrating diagnoses between the two levels of healthcare.

Our analysis revealed that certain patient-related variables significantly increase the risk of stroke, including age, gender, CHA_2_DS_2_Vasc Score, dyslipidemia, insulin-dependent diabetes, noninsulin-dependent diabetes, ischemic heart disease with angina, cerebrovascular disease, acute myocardial infarction, hypertension with complications, and heart failure. However, our findings did not verify a higher likelihood of stroke in patients with chronic alcohol abuse. Reddiess et al. (25) suggest that patients with AF and low to moderate alcohol intake are not associated with an increased risk of stroke and other cardiovascular events (25). In contrast, Lee et al. (20) found an association between current alcohol consumption and an increased risk of ischemic stroke in patients with newly diagnosed AF. Current drinkers with a mild amount of alcohol consumption showed a significantly higher risk of ischemic stroke, with a linear dose-response relationship between the amount of current alcohol intake and the risk of ischemic stroke (20). Since our study did not quantify alcohol consumption and relied on codifying the problem as “chronic alcohol abuse,” there may be an information bias.

A review of existing evidence shows that heart failure independently increases the risk of stroke, regardless of AF, through various mechanisms, mainly thromboembolism (26). This aligns with our results, and a multicenter observational study in seven European countries also confirmed risk factors for embolic events in anticoagulated AF patients, such as prior stroke, older age, and heart failure (16).

Regarding gender, several studies have indicated that the female sex is an independent risk factor in patients with AF. Our results also found that females were significantly associated with a higher likelihood of experiencing an event, in line with the existing literature (27–29). This may be attributed to post-menopausal vascular changes related to the reduction in estrogen that affects lipid metabolism, increases the risk of left ventricular remodeling and hypertension, and leads to an increase in inflammatory and procoagulant markers, all contributing to thromboembolism (27, 29).

The CHA_2_DS_2_-VASc Score is a point-based system used to stratify the risk of stroke in AF patients, with higher scores indicating greater stroke risk (30). Multiple studies have shown increased stroke and mortality with an increasing CHA_2_DS_2_-VASc Score (30). Additionally, diabetes independently increases the risk of stroke in patients with AF by 1.7-fold, with other studies noting an absolute stroke rate of 2.0–3.5% per year without other risk factors in non-anticoagulated AF patients (31).

In a large cohort study using the Danish National Patient Registry, coronary artery disease (defined as obstructive (≥50%) coronary stenosis in ≥1 coronary vessel or non-obstructive coronary stenosis in ≥2 coronary vessels) was found to confer a 29% increased risk of ischemic stroke among patients with AF, suggesting that coronary artery disease was an independent risk factor for ischemic stroke (32). Polzin et al. (33, 34) showed that the factor IIa inhibition by dabigatran might increase the risk of myocardial infarction by enhancing platelet reactivity through several potential mechanisms. In contrast, factor Xa inhibition might reduce the risk of myocardial infarction. In another review, they found translational studies that identified different prothrombogenic non-canonical effects under FIIa inhibitor treatment, yielding increased platelet reactivity. In contrast, different non-canonical mechanisms resulting in reduced platelet reactivity and thrombus formation in patients treated with FXa inhibitors were described. Thus, these results may explain why the variables ischemic heart disease with angina and acute myocardial infarction are related to increase the risk of stroke.

Hypertension also contributes to stroke, with event rates increasing at systolic blood pressure (SBP) levels of 140 mmHg and above. However, controlled hypertension with a mean SBP < 140 mmHg is associated with a lower stroke risk than patients with poorly controlled hypertension (35).

Interestingly, a study conducted using the electronic database of Clalit Health Services in Israel showed no association between LDL-C levels and incident ischemic stroke within each CHA_2_DS_2_VASc Score group, even after multivariate adjustment (36). Conversely, another study indicated that LDL levels above 1.5 mmol/L were independently associated with higher stroke rates in patients with AF, while statins were associated with lower stroke rates independent of anticoagulation. This suggests that LDL measurements may improve stroke risk stratification in AF (37).

These findings align with our results, which demonstrate that dyslipidemia increases the risk of stroke in patients with AF.

Regarding limitations, it is essential to acknowledge that generalizing these results to similar populations is limited, as the data are specific to a population in the north of Portugal. Furthermore, the statistically significant associations presented in this study exhibit a small effect size, possibly due to the large sample size. Another limitation is related to stroke coding in ICPC-2, where it is impossible to differentiate ischemic stroke from hemorrhagic stroke.

One of the strengths of our study lies in the large number of patients with AF included and the 3-year follow-up conducted to assess the occurrence of events. Additionally, considering all NOAC dispensations made by patients, regardless of whether a family doctor issued the prescription, a hospital doctor from the National Health Service, or a doctor from a private institution, adds to the robustness of the data and provides a more realistic perspective on compliance with NOAC usage. These results present novel findings in studies with real-world data, warranting further in-depth analysis to strengthen the reliability of these results in future research.

## Conclusion

This study provides important insights from real-world data concerning patients with AF treated with NOACs in the Northern Region of Portugal. On the one hand, we observed variations between patients with AF receiving each of the four NOACs and several studied variables. On the other hand, approximately 10.2% of AF patients on NOAC anticoagulation experienced a stroke highlights the need for a thorough analysis of prescription appropriateness and adherence to anticoagulation in this patient population.

Another crucial finding from our study pertains to the probability of stroke among the four NOACs. Our analysis demonstrates that patients treated with apixaban and dabigatran exhibited significantly higher odds of experiencing a stroke than those treated with rivaroxaban. Additionally, among patients of the same age, gender, and CHA_2_DS_2_Vasc Score, apixaban was significantly associated with an increased likelihood of thrombotic events compared to rivaroxaban.

## Data availability statement

The raw data supporting the conclusions of this article will be made available by the authors, without undue reservation.

## Ethics statement

The studies involving humans were approved by the Health Ethics Committee and Data Protection Officer of the Northern Regional Health Administration. The studies were conducted in accordance with the local legislation and institutional requirements. Written informed consent for participation was not required from the participants or the participants’ legal guardians/next of kin in accordance with the national legislation and institutional requirements.

## Author contributions

SP: Investigation, Writing – original draft. AT: Conceptualization, Writing – review and editing. TH: Methodology, Writing – review and editing. HM: Software, Writing – review and editing. CM: Supervision, Writing – review and editing.
