# Supplementary material for: AF-React study: Prevalence of thrombotic events in patients with atrial fibrillation receiving NOACs – real-world data analysis from northern Portugal primary healthcare
Source: Front Med (Lausanne). 2024 Apr 12;11:1273304. doi: 10.3389/fmed.2024.1273304 (PMC11046733; doi:10.3389/fmed.2024.1273304)
Supplement: Supplementary file 1 [file Table_1.DOCX]

**Appendix A**

|  | **Simple Logistic Regression** | | |
| --- | --- | --- | --- |
|  | **OR**  **95% CI** | | ***p-value*** |
| **Drug** |  |  | |
| *Rivaroxaban* | *Reference* | | |
| *Apixaban* | 1.38 [1.25; 1.53] | **<0.001** | |
| *Dabigatran* | 1.26 [1.13; 1.40] | **<0.001** | |
| *Edoxaban* | 1.16 [0.92; 1.45] | 0.207 | |
| **Age** | 1.04 [1.03; 1.04] | **<0.001** | |
| **Gender** |  |  | |
| *Male* | *Reference* | | |
| *Female* | 1.11 [1.02; 1.21] | **0.013** | |
| **Profissional situation** |  |  | |
| *Active or student* | *Reference* | | |
| *Retired* | 1.86 [1.66; 2.08] | **<0.001** | |
| *Not active or Unknown* | 1.21 [1.00; 1.46] | **0.048** | |
| **CHA_2_DS_2_-VASc** | 3.63 [3.47; 3.80] | **<0.001** | |
| **RCV 2016** | 4.75 [4.18; 5.40] | **<0.001** | |
| **RCV 2017** | 9.24 [7.74; 11.0] | **<0.001** | |
| **GFR 2016** | 1.00 [1.00; 1.00] | 0.766 | |
| **GFR 2017** | 1.00 [1.00; 1.00] | 0.353 | |
| **GFR 2018** | 1.00 [1.00; 1.00] | **0.197** | |
| **Comorbidities** |  |  | |
| *Chronic alcohol abuse, P15* | 0.87 [0.70; 1.09] | 0.232 | |
| *Tobacco abuse, P17* | 0.71 [0.57; 0.88] | **0.002** | |
| *Lipid disorder, T93* | 1.25 [1.15; 1.36] | **<0.001** | |
| *Atherosclerosis/PVD, K92* | 0.46 [0.40; 0.52] | **<0.001** | |
| *Diabetes insulin dependent, T89* | 1.56 [1.20; 2.01] | **<0.001** | |
| *Diabetes non-insulin dependent, T90* | 1.11 [1.01; 1.22] | **0.033** | |
| *Coronary heart disease ischaemic heart disease with angina, K74* | 1.17 [0.97; 1.42] | **0.107** | |
| *Ischaemic heart disease w/o angina, K76* | 1.07 [0.87; 1.31] | 0.532 | |
| *Cerebrovascular disease, K91* | 7.02 [6.13; 8.04] | **<0.001** | |
| *Acute myocardial infarction, K75* | 1.73 [1.42; 2.11] | **<0.001** | |
| *Hypertension complicated, K87* | 2.86 [2.62; 3.11] | **<0.001** | |
| *Hypertension uncomplicated, K86* | 0.52 [0.48; 0.57] | **<0.001** | |
| *Heart failure, K77* | 1.26 [1.15; 1.39] | **<0.001** | |

Legend: Simple Logistic Regression analysis of the association between the several variables and the thrombotic event risk (ICPC-2, K89 or K90)
